# Phenotypic and genetic characterization of *Piscirickettsia salmonis* from Chilean and Canadian salmonids

**DOI:** 10.1186/s12917-016-0681-0

**Published:** 2016-03-15

**Authors:** Alexander Otterlei, Øyvind J. Brevik, Daniel Jensen, Henrik Duesund, Ingunn Sommerset, Petter Frost, Julio Mendoza, Peter McKenzie, Are Nylund, Patricia Apablaza

**Affiliations:** Fish Diseases Research Group, Department of Biology, University of Bergen, P.O. 7803, N-5020 Bergen, Norway; Cermaq Group AS, Dronning Eufemias gate 16, P.O. Box 144 Sentrum, N-0102 Oslo, Norway; MSD Animal Health, Thormølensgt. 55, 5008 Bergen, Norway; Cermaq Chile, Diego Portales 2000, piso 10, Puerto Montt, Chile; Cermaq Canada, 203 - 919 Island Highway, Campbell River, BC V9W 2C2 Canada

**Keywords:** *P. salmonis*, Phenotyping, Genotyping, 16S rDNA-ITS, Housekeeping genes, Phylogeny, MLST

## Abstract

**Background:**

The study presents the phenotypic and genetic characterization of selected *P. salmonis* isolates from Atlantic salmon and rainbow trout suffering from SRS (salmonid rickettsial septicemia) in Chile and in Canada. The phenotypic characterization of the *P. salmonis* isolates were based on growth on different agar media (including a newly developed medium), different growth temperatures, antibiotics susceptibility and biochemical tests.

**Results:**

This is the first study differentiating Chilean *P. salmonis* isolates into two separate genetic groups. Genotyping, based on 16S rRNA-ITS and concatenated housekeeping genes grouped the selected isolates into two clades, constituted by the Chilean strains, while the Canadian isolates form a branch in the phylogenetic tree. The latter consisted of two isolates that were different in both genetic and phenotypic characteristics. The phylogenies and the MLST do not reflect the origin of the isolates with respect to host species. The isolates included were heterogeneous in phenotypic tests.

**Conclusions:**

The genotyping methods developed in this study provided a tool for separation of *P. salmonis* isolates into distinct clades. The SRS outbreaks in Chile are caused by minimum two different genetic groups of *P. salmonis.* This heterogeneity should be considered in future development of vaccines against this bacterium in Chile. Two different strains of *P. salmonis*, in regards to genetic and phenotypic characteristics, can occur in the same contemporary outbreak of SRS.

**Electronic supplementary material:**

The online version of this article (doi:10.1186/s12917-016-0681-0) contains supplementary material, which is available to authorized users.

## Background

The Chilean aquaculture industry has constantly faced problems with *Piscirickettsia salmonis*, which causes salmonid rickettsial septicemia (SRS) [1]. This disease causes high mortalities in several salmonids species Atlantic salmon (*Salmo salar*), Rainbow trout (*Onchorhyncus mykiss*), Coho salmon (*Onchorhyncus kisutch*), and was responsible for 90 % of the total use of antibiotics in Chile in 2014 [2]. *P. salmonis* has been detected in several countries including Norway, Canada, Scotland, Ireland and Chile [3, 4], but opposed to the situation in Chile, SRS is considered manageable in these countries.

The present knowledge on *P. salmonis* is restricted in several aspects such as characterization (phenotypical and genetic), geographical distribution, host specificity, presence of natural reservoirs and transmission routes. Although several vaccines are available [5–7] none of them are able to induce complete protection against SRS. This lack of protection suggests that more basic knowledge is needed about the biology of *P. salmonis*.

*P. salmonis* is a Gram-negative, predominantly coccoid, aerobic, non-encapsulated, and highly fastidious bacterium of approx. 0.5–1.5 μm diameter [3]. The pathogen was first described as an obligatory intracellular bacterium and has traditionally been cultured on Chinook salmon embryos cells (CHSE-214) and others fish cell lines [8]. In 2008 it was shown that *P. salmonis* could be grown on artificial cell-free media [9–11]. However, work at our laboratory has shown that not all isolates growth well at these media, hence, we developed a new improved medium for isolation and culturing of *P. salmonis* strains.

This study present new basic knowledge of *P. salmonis* isolated from sub-acute, acute and chronic outbreaks of SRS. These isolates are compared to the type strain, LF-89. The new knowledge presented could form a better basis for development of efficient strategies for control and treatment of SRS, and possibly a new basis for future development of vaccines against *P. salmonis.*

## Methods

### Collection of *P. salmonis*

**Fig. 1:**
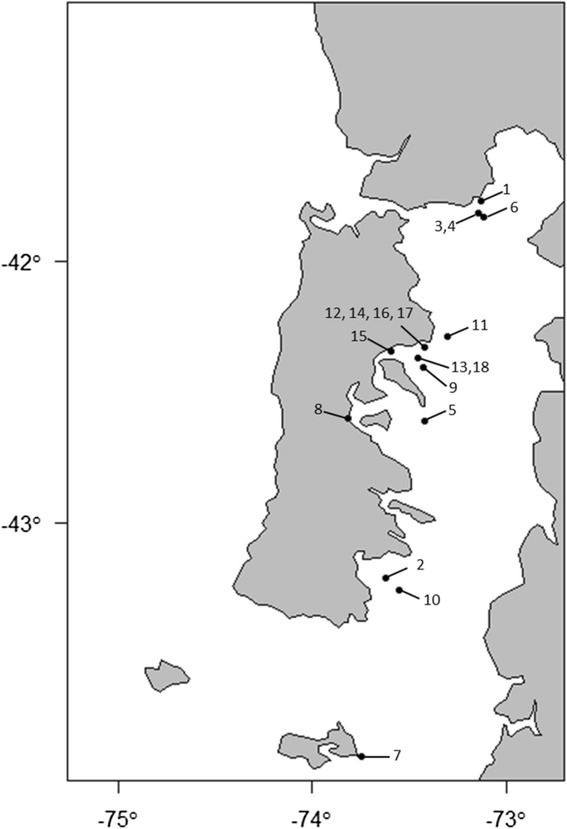
Geographical location of the Chilean farms from were *P. salmonis* isolates were obtained. The number correspond to isolates: 1 = LF-89, 2 = Ch2-As-I, 3 = Ch3-Rt-L, 4 = Ch4-Rt-L, 5 = Ch5-As-I, 6 = Ch6-Rt-L, 7 = Ch7-As-L, 8 = Ch8-Rt-K, 9 = Ch9-As-na, 10 = Ch10-As-I, 11 = Ch11-As-I, 12 = Ch12-As-I, 13 = Ch13-As-I, 14 = Ch14-As-I, 15 = Ch15-As-I, 16 = Ch16-As-I, 17 = Ch17-As-I, and 18 = Ch18-As-I

The isolates of *P. salmonis* were obtained in 2011 and 2012 from Atlantic salmon and Rainbow trout suspected or diagnosed with SRS. The Chilean isolates, were obtained from a pool of homogenized fish tissues (kidney, spleen, liver, brain). The Canadian isolates were collected from the same site during a single outbreak of SRS, and from kidney. All the isolates were sent to the Fish Diseases Research Group at the University of Bergen where they were sub-cultured on CHAB agar [10] and stored at −80 °C for later characterization. An overview of all the isolates included in the study is presented in the Table 1. The geographical origins of the isolates are presented in Fig. 1. The *P. salmonis* type isolate (LF-89) was also included in the study.

**Table 1:** Data for all the selected *P. salmonis* isolates included in the study

Isolate code	Country	County (Region)	Sampling date	Mortality (%)	Host	Sample tissue	Clinical condition
LF-89	Chile	Puerto Montt (X)	1989	na	Coho salmon	kidney	na
Ch2-As-I	Chile	Chiloé Sur (X)	08.08.2012	7,8	Atlantic salmon	k-l-sp-b	acute
Ch3-Rt-L	Chile	Calbuco (X)	03.10.2012	6,7	Rainbow trout	lession	sub-acute
Ch4-Rt-L	Chile	Calbuco (X)	05.10.2012	6,7	Rainbow trout	lession	sub-acute
Ch5-As-I	Chile	Chiloé Centro (X)	18.07.2012	5,1	Atlantic salmon	k-l-b	sub-acute
Ch6-Rt-L	Chile	Calbuco (X)	10.08.2012	13,2	Rainbow trout	lession	sub-acute
Ch7-As-L	Chile	Aysén (XI)	na	na	Atlantic salmon	muscle	chronic
Ch8-Rt-K	Chile	Chiloé Centro (X)	17.06.2011	1,9	Rainbow trout	kidney	sub-acute
Ch9-As-na	Chile	Chiloé Centro (X)	27.03.2012	2,4	Atlantic salmon	na	sub-acute
Ch10-As-I	Chile	Chiloé Sur (X)	24.07.2012	21,5	Atlantic salmon	k-l-sp-b	acute
Ch11-As-I	Chile	Chiloé Centro (X)	04.05.2012	2,2	Atlantic salmon	k-l-b	sub-acute
Ch12-As-I	Chile	Chiloé Centro (X)	07.05.2012	14,8	Atlantic salmon	k-l-b	acute
Ch13-As-I	Chile	Chiloé Centro (X)	18.04.2012	3,6	Atlantic salmon	k-l-sp-b	chronic
Ch14-As-I	Chile	Chiloé Centro (X)	13.01.2012	14,8	Atlantic salmon	k-sp	acute
Ch15-As-I	Chile	Chiloé Centro (X)	23.08.2012	5,4	Atlantic salmon	k-l-b-sp	sub-acute
Ch16-As-I	Chile	Chiloé Centro (X)	June 2012	14,8	Atlantic salmon	k-l-b	acute
Ch17-As-I	Chile	Chiloé Centro (X)	23.05.2012	14,8	Atlantic salmon	k-l-b	acute
Ch18-As-I	Chile	Chiloé Centro (X)	22.06.2012	3,6	Atlantic salmon	k-l-b-sp	chronic
Ca19-As-I	Canada	British Columbia	11.12.2012	<0,03	Atlantic salmon	kidney	chronic
Ca20-As-I	Canada	British Columbia	11.12.2012	<0,03	Atlantic salmon	kidney	chronic

Codes indicates the country of isolation, Chile (Ch), Canada (Ca); number of isolate (1–20); fish species, Atlantic salmon (As), Rainbow trout (Rt), Coho salmon (Cs); and tissue, kidney (K), liver (l), spleen (sp) and brain (b). The samples were obtained from a pool of internal organs. The codes are: I from internal lesions, L from external lesions, and K from kidney. Sub-acute and acute clinical conditions frequently present hemorrhages in the brain, splenomegaly and extensive congestion particularly in the swimbladder. In chronic conditions typical findings are whitish nodules in the liver which becomes brownish and finally progress as granuloma. Mostly in Atlantic salmon and rainbow trout, the dermis present speckle erosions which progress to pustules and finally caverns. It has also seen splenomegaly and hepatomegaly; pericarditis with fibrin deposits in Atlantic salmon and rainbow trout. In Coho salmon the fibrin deposits are located mostly internally in the abdomen. Mortality percentage is described as percentage of dead fish due to SRS, from necropsy findings, in one production cycle. In most of the farms antimicrobials were used for treatment. Na: information not available

This study followed the recommendations of the Norwegian Animal Welfare Act (01.01.2010) and the work was done under the regulations given by the Norwegian Food Safety Authority. The bacterial samples were obtained from the fish after they has been anaesthetized by a blow to the head and killed by instantly decapitation. This procedure complies with the Norwegian fish welfare regulations.

### New growth medium

To improve the growth of *P. salmonis* on solid media, a new optimized SRS blood agar (SRS-BA) was developed. The composition of this agar was 40 g of TSA (BD, Difco); 20 g of Red Sea Salt (RSS) (Red Sea, USA); 50 ml of defibrinated sheep blood (DSB) (Oxoid Limited, UK); 1 g of L-cysteine (Sigma-Aldrich); 5 g of D-glucose (Sigma-Aldrich); 50 ml of fetal bovine serum (FBS) (Thermo Scientific Hyclone, USA); 0.2 mM ferric nitrate (Sigma-Aldrich) and reverse osmosis water (RO) to a final volume of 1000 ml. RSS was chosen to simulate natural sea water composition. RO water, TSA and RSS were autoclaved at 121 °C for 15 min. DSB was added after cooling from autoclaving. Subsequently, the agar was cooled down to 50 °C and L-cysteine, D-glucose, FBS and ferric nitrate were added. The pH was adjusted to 6.8 ± 0.2.

### Phenotypic characterization

Colonies > 1 mm were used for morphological characterization and bacteria from these colonies were Gram-stained for the description of cell morphology and other phenotypic characteristics.

Testing of different growth media was performed using two colonies from each isolate streaked on the following media: SRS-BA, Austral-TSHem [11], CHAB [10], CHAB with 0.2 mM Fe, blood agar (BA), BA with 2 % NaCl, marine agar (MA) and tryptone-yeast extract-salts with glucose agar (FLPA) [12] and incubated at 19 °C for 14 days. The temperature range for bacterial growth measured as number of colonies and colony size was tested using three colonies streaked out on SRS-BA and incubated at 8, 11, 16, 19, 22, and 25 °C. The growth was recorded at days 3, 7, 10 and 14 in both tests.

Test of the susceptibility to antimicrobial drugs was performed by the agar diffusion method using tablets as described by Justesen et al. [13], with some modifications. 19 °C was used for incubation, as this is within the common optimum temperature range of all isolates. The inhibition zone was measured after 10 days of incubation, to ensure sufficient growth. The antimicrobial sensitivity was tested for all the isolates against: streptomycin (10 μg); oxytetracycline (30 μg); penicillin (1 unit); ceftazidime (30 μg); ampicillin (2 μg); and florfenicol (30 μg). The inhibition zone was measured, in millimeters, from the tablet border towards to all the extension of the inhibition area.

Indole and oxidase tests were performed with BBL™ DrySlide™ (BD BBL™, U.S.A) kits. The cefinase test using BBL™ Cefinase™ Paper Disc (BD BBL™, U.S.A) was used to determine the production of β-lactamases. Catalase was detected by adding a drop of hydrogen peroxide 3 % (Sigma-Aldrich, Germany) to a microscope slide before transferring a small amount of bacteria onto the H_2_O_2_ solution. The API ZYM galleries (BioMérieux, U.S.A) for the detection of bacterial enzymes were used according to the manufacturer’s protocol, except that the incubation temperature was set at 19 °C and the incubation time at 24 h. A 6.0 McFarland standard, as recommended by BioMérieux, was made in advance for turbidity comparison. H_2_S production was done using hydrogen Sulfide Test Strips (Sigma-Aldrich, Switzerland). All the phenotypical and biochemical tests were performed in triplicate, with the exception of the antimicrobial susceptibility analysis, which was done in duplicate.

### Genetic characterization

The DNA extraction from pure cultures of all *P. salmonis* was performed with an E.Z.N.A Tissue DNA kit. The quality of DNA was tested using NanoDrop (Saveen Werner, Life Science) before storing at −20 °C.

PCR, purification and visualization of products by gel electrophoresis, and sequencing were performed as described by Apablaza et al. [14]. Sequencing of the 20 isolates was performed using specific primers for 16S rDNA and ITS, and for the housekeeping genes, HK (dnaK, groEL, tbpB, mltB, ospA, radA, airA, bax, tnpA, elfP). The primers are presented in Table 2. The Vector NTI® v9.0 Software package (Invitrogen) was used for sequence assembling and alignment. Manual adjustments and verification of reading frames for the protein coding genes were conducted in GeneDoc (Karl Nicholas ©2000). The evolutionary analysis and calculation of the best fit model for the 16S rDNA-ITS dataset (2191 base pairs) was conducted in MEGA6 [15]. The maximum likelihood method using the Hasegawa-Kishino-Yano [16] model with discrete Gamma distribution was applied.

**Table 2:** Primers used for PCR analysis in the present study

Target gene	Primer	Direction	Sequence (5´–3´)	Reference
16 s rDNA	Eug B27F	Fwd	AGAGTTTGATCMTGGCTCAG	[25]
	Eug A1518R	Rev	AAGGAGGTGATCCANCCRCA	[25]
ITS	SRS-ITS/F	Fwd	GTACACACCGCCCGTCACAC	Present study
	SRS-ITS/R	Rev	CCTCACGGTACTAGTTCACTATCGG	Present study
dnaK	SRS-dnaK/F2	Fwd	CCGTGTCGTGTGGCGCTAAAA	Present study
	SRS-dnaK/R2	Rev	TTGAGATTGAGCCTGCTCCGC	Present study
	SRS-dnaK3/F1	Fwd	CCGCGTGTGATTGAGAGTGC	Present study
	SRS-dnaK3/R1	Rev	CGTCATCACCCCACCCATGG	Present study
groEL	SRS-groEL/F1	Fwd	CTTCGGTACCGGTTCCCGTC	Present study
	SRS-groEL/R1	Rev	TCTTGCAGTTTCTCGCGGTCG	Present study
	SRS-groEL/F2	Fwd	GTGAAGCTCTGGCAACACTCGTC	Present study
	SRS-groEL/R2	Rev	AGGAAGCTCTGCAACCATCGC	Present study
tbpB	SRS-tbpB/F1	Fwd	AACTGGGCAGGCGTCACTGTT	Present study
	SRS-tbpB/R1	Rev	CGGCGCGTCTCTAATGTTCG	Present study
	SRS-tbpB2/F2	Fwd	CCAAGCTGGATCACCGCCAT	Present study
	SRS-tbpB2/R2	Rev	AAAGATAGGCCCAGCCACGC	Present study
mltB	SRS-mltB/F	Fwd	ACCACTCACGCGGCATCTAA	Present study
	SRS-mltB/R	Rev	ACTCAAATCATACACCGCCATTGCA	Present study
ospA	SRS-ospA/F	Fwd	AGCCGTCAAGAAGTCGGAGCT	Present study
	SRS-ospA/R	Rev	TGCCAACGACCATCCGCTTG	Present study
radA	SRS-radA/F1	Fwd	ATCAGTCGCCAGCCTGTTGG	Present study
	SRS-radA/FR1	Rev	GTCCTCGTTGCACTGGACGA	Present study
airA	SRS-air/F1	Fwd	GGGTGCGTCCGGGGATTATG	Present study
	SRS-rairA/R1	Rev	TAAGGTGCACGCAGTGGCAT	Present study
bax	SRS-bax/F1	Fwd	TCAAGGGATCTGGGAAGTGCT	Present study
	SRS-bax/R1	Rev	ACCACTGCCTATCTTGCTCAACA	Present study
tnpA	SRS-tnpA/F1	Fwd	ACCTGTTAAGTTCTCGGCCATT	Present study
	SRS-tnpA/R1	Rev	AGCCTTCACAAATGTCAACAAGTGA	Present study
elfP	SRS-elfP/F	Fwd	GCCACKGCTAATTCAGCAA	Present study
	SRS-elfP/R	Rev	STGGAATGGTCAGCCACYT	Present study

The software KAKUSAN4 was used for construction of concatenated sequences of the 10 HK genes (8969 base pairs), calculation of substitution rate, and the best fit model for the individual loci and codon positions [17]. The data were exported into an MrBays-block (V. 3.2.2 x86) for analysis. The phylogenetic analysis was performed with Proportional Codon, Proportional model and a mcmc of 40 000 000 generations. The phylogram was constructed using TreeAnotator and viewed in FigTree [18]. A statistics report of percentage identity (PID) and average nucleotide identity (ANI) was made in GeneDoc (Karl Nicholas ©2000).

The multi locus sequence typing (MLST) method was applied as described by Apablaza et al. [14]. Briefly, the different allele types (AT) within the 10 HK were used to create the sequence types (ST). Based on the different STs, a data matrix from the 18 isolates of *P. salmonis* was exported as a nexus file into PAUP 4.0. A dendrogram was constructed using neighbor-joining (NJ) distance method.

All the nucleotide sequences were deposited in the GenBank database. Accession numbers are presented in Additional file 1: Table S1.

## Results

### Phenotypic characterization

The colonies grown on SRS-BA were slightly convex, grey-white, shiny, and centrally opaque with translucent, slightly undulating margins. At the microscopic level, the cells were Gram-negative, coccoid shaped, measuring from 0.4 to 1.8 μm in diameter. No distinctive colony or cell morphology differences were observed among the isolates.

**Fig. 2:**
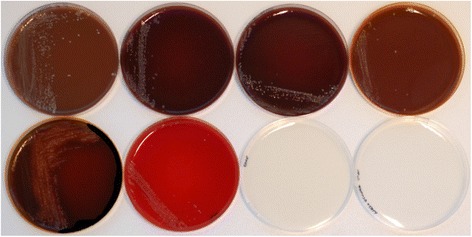
Isolate Ch14-As-I cultured in eight different growth media: from upper left to the right, SRS-BA, CHAB, CHAB supplemented with ferric nitrate; Austral-TSHem; BA w/2 % NaCl, BA, MA, and FLPA

The results of the growth at the different media are presented in Table 3. The minority of the isolates were classified as ‘fastidious’ (LF-89, Ch5-As-I, Ch6-Rt-L, and Ca20-As-K) while the rest of the isolates were defined as ‘less fastidious’. Five of the isolates (Ch3-Rt-L, Ch4-Rt-L, Ch5-As-I, Ch6-Rt-L and Ca20-As-I) grew on SRS-BA only. No growth was recorded on MA and FLPA media. The isolate Ch15-G2-As-I produced the highest number of colonies in SRS-BA. Ch14-As-I induced α- (dark green/brown discoloration around colonies) and β-hemolysis (medium translucence under the colonies) on BA with 2 % NaCl. This isolate grew on all the tested media, with exception of MA and FLPA (Fig. 2).

**Table 3:** Summary of growth of *P. salmonis* isolates on different culture media

Isolate	SRS-BA	CHAB	CHAB w/Fe	Austral-TSHem	BA	BA w/2 % NaCl
Ch11-As-I	+++	+++	+++	++	++	+++
Ch7-As-L	+++	+++	+++	++	++	++
Ch14-As-I	+++	++	++	++	++	+++
Ch9-As-na	+++	+++	+++	++	+	+
Ch13-As-I	+++	++	++	++	++	+++
Ch17-As-I	++	++	+	+	++	++
Ch2-As-I	+++	++	++	+	++	++
Ch12-As-I	+++	++	++	+	+	++
Ch15-As-I	+++	+	+	W	+	+
Ch10-As-I	++	++	++	+	+	++
Ch8-Rt-K	+++	+	+	+	+	+
Ch18-As-I	+++	+	+	+	-	+
Ca19-As-I	+++	++	++	-	+	+
Ch16-As-I	++	+	+	-	+	++
LF-89	+++	++	++	-	+	+

Symbols: (−), no growth; (+), scant growth; (++), moderate growth; (+++), vigorous growth; and (w), weak growth. Isolates Ch3-Rt-L, Ch4-Rt-L, Ch5-As-I, Ch6-Rt-L, and Ca20-As-I were grew only in SRS-BA

The maximum temperature range for the isolates growth was from 8 to 25 °C on SRS-BA (data not shown). Ch2-As-1, Ch7-As-L, Ch8-Rt-K, Ch12-As-I, Ch18-As-I, and Ca19-As-K had the ability to grow at 25 °C, while the type strain LF-89 did not grow at this temperature. After three days of incubation all isolates showed growth at 16, 19 and 22 °C. The optimum temperature for maximum growth was in the range 19–22 °C for the less fastidious isolates (including Ca19-As-K), and 16 to 19 °C for the remaining strains, including Ca20-As-K (Table 4).

**Table 4:** Optimum growth temperature for the *P. salmonis* isolates

Isolate	Optimum growing temp (°C)
LF-89	16–19
Ch2-As-I	16–19
Ch3-Rt-L	16–19
Ch4-Rt-L	16–19
Ch5-As-I	16–19
Ch6-Rt-L	16–19
Ca20-As-K	16–19
Ch7-As-L	19–22
Ch8-Rt-K	19–22
Ch9-As-na	19–22
Ch10-As-I	19–22
Ch11-As-I	19–22
Ch12-As-I	19–22
Ch13-As-I	19–22
Ch14-As-I	19–22
Ch15-As-I	19–22
Ch16-As-I	19–22
Ch17-As-I	19–22
Ch18-As-I	19–22
Ca19-As-K	19–22

The study of the antibiotic sensitivity of the isolates with respect to different antibiotics shows large variation in the inhibition zones. The strain Ch3-Rt-L seems to be resistant to the most of the antibiotics tested (Table 5). All isolates were sensitives to oxytetracycline, while florfenicol yielded the largest inhibition zones with the exception of above mentioned strain. Ch10-As-I had the largest inhibition zones in all antibiotics with the exception of penicillin.

**Table 5:** Antibiotic inhibition zones (mm) of the *P. salmonis* isolates

Isolate	Streptomycin	Oxytetracycline	Penicilin	Ceftazidime	Ampicilin	Florfenicol
LF-89	5	23	12	15,5	19,5	24
Ch2-As-I	0,5	0,5	1,5	10	3	14,5
Ch3-Rt-L	3,5	8,5	0,5	0,5	2,5	4
Ch4-Rt-L	2	18	6,5	17,5	14	19
Ch5-As-I	1	17	12	16,5	12,5	19,5
Ch6-Rt-L	0	16,5	13	15,5	13	18
Ch7-As-L	0	10	10	13,5	10,5	14,5
Ch8-Rt-K	1	21	2	13,5	13,5	24,5
Ch9-As-na	2	25	7	16	9,5	24
Ch10-As-I	32,5	27,5	1,5	18,5	22	23,5
Ch11-As-I	1	21	4	5	7,5	19
Ch12-As-I	0,5	17	9,5	12,5	1,5	22
Ch13-As-I	0,5	22	2,5	10	10	25
Ch14-As-I	0	22	9,5	11,5	11,5	24,5
Ch15-As-I	1	20	6	12	9	20,5
Ch16-As-I	0	3,5	6,5	12,5	8	17,5
Ch17-As-I	0,5	24,5	5,5	11	14,5	23
Ch18-As-I	2,5	20	0	10	1,5	15,5
Ca19-As-I	2	17	21,5	22	25	24,5
Ca20-As-I	2,5	24	13	13,5	15	27,5

Indole and oxidase tests were negative for all of the isolates, while catalase and cefinase tests were positive.

In the APY ZYM galleries, the results which differentiate the isolates are shown in Table 6. The tests were negative for all the isolates in the remaining enzymes included in the gallery. The Canadian isolate Ca19-As-I and the Chilean Ch8-Rt-K were the only ones positive for the enzyme esterase lipase and esterase, respectively. In addition, the isolate Ch14-As-I gave a positive reaction for β-galactosidase and α-glucosidase while the rest of the isolates were negative.

**Table 6:** *P. salmonis* differential enzymatic reactions in API ZYM galleries

Isolate	AP	E	EL	LA	VA	CA	AP	NP
LF-89	W	W	W	+	+	W	+	+
Ch2-As-I	W	W	W	+	+	−	W	W
Ch3-Rt-L	W	W	W	+	+	−	W	+
Ch4-Rt-L	W	W	W	+	+	−	W	+
Ch5-As-I	W	W	W	+	+	−	W	W
Ch6-Rt-L	W	W	W	+	+	W	+	+
Ch7-As-L	+	W	W	+	W	W	+	W
Ch8-Rt-K	+	+	W	W	W	−	+	W
Ch9-As-na	+	W	W	+	+	−	+	W
Ch10-As-I	+	W	W	+	+	+	+	+
Ch11-As-I	+	W	W	+	+	−	+	W
Ch12-As-I	+	W	W	+	+	−	+	+
Ch13-As-I	+	W	W	W	W	+	+	W
Ch14-As-I	+	W	W	+	+	−	+	+
Ch15-As-I	+	W	W	+	+	−	+	+
Ch16-As-I	+	W	W	+	+	−	+	W
Ch17-As-I	+	W	W	+	+	−	+	+
Ch18-As-I	+	W	W	+	+	−	+	+
Ca19-As-K	+	W	+	+	+	−	+	+
Ca20-As−K	+	W	W	+	+	−	+	+

W: weak reaction; +: positive reaction; −: negative reaction

**Table 7:** Overview of the number of variable nucleotide and putative aminoacids positions in the alignment of 16S rDNA, ITS and the ten housekeeping genes of *P. salmonis* included

Gene	N	No. Nucleotides	Variable positions	%	No. Amino acids	Variable positions	%
16S	18	1433	14	1,0	-	-	-
ITS	18	765	34	4,4	-	-	-
dnaK	18	1684	66	3,9	560	3	0,5
groEL	18	1454	73	5,0	484	10	2,1
tbpB	18	1879	95	5,1	626	26	4,2
mltB	18	843	20	2,4	280	5	1,8
ospA	18	364	14	3,8	121	3	2,5
radA	18	906	37	4,1	301	5	1,7
airA	18	407	21	5,2	135	6	4,4
bax	18	612	18	2,9	203	9	4,4
tnpA	18	356	20	5,6	118	5	4,2
elfP	18	473	26	5,5	157	0	0,0
MLST	216	11176	438	4,1	2985	7,2	2,6

Symbols: N: number of *P. salmonis* sequences

### Genetic characterization

**Fig. 3:**
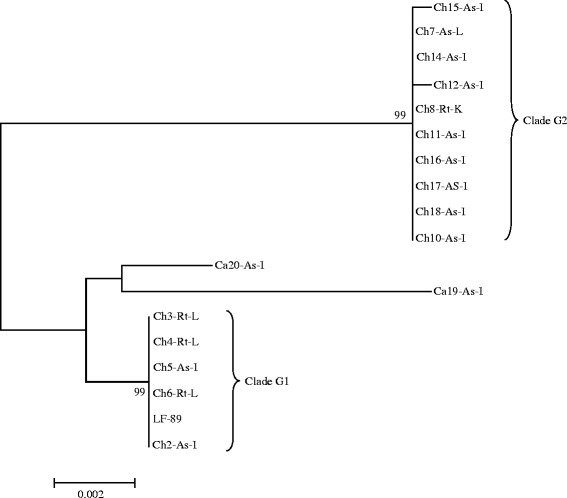
The phylogenetic relationships of the 18 isolates of *P. salmonis*, based in the analysis of 2198nt within 16S rDNA-ITS genes. The phylogenetic tree was obtained by Mega 6.05 software. The codes for all the isolates are explained in the Table 1

**Fig. 4:**
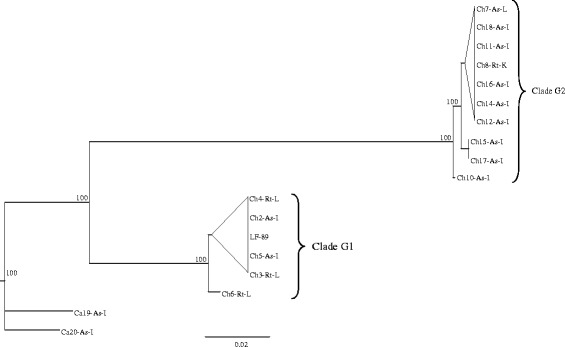
The genetic relationships of the 18 isolates of *P. salmonis*, based in the analysis of concatenated HK. The phylogenetic three was obtained by the mrBayes software. The codes for all the isolates are explained in the Table 1

Phylogenetic analysis of the 18 partial (2198 bp) sequences of the 16S rDNA-ITS genes identified two clades (G1 and G2) as shown in the Fig. 3. The Canadian isolates are distinct and belong to the same branch in the phylogenetic tree. The analysis of the genetic relationships of the concatenated HKs is presented in Fig. 4. After several PCR attempts it was not possible to obtain all the expected PCR products for the isolates Ch13-As-I and Ch9-As-na, therefore they were not includes in the genetic analysis.

Comparison of 10 housekeeping sequences from the isolates, included in the MLST scheme, revealed 438 variable nucleotide positions across 11176 nucleotides (nts) (Table 7). The highest percentage of variable nucleotide positions (5.6 %) was seen in the tnpA locus and the lowest (2.4 %) in mltB. The putative amino acid sequence showed variation in 7.2 positions of a total of 2985 amino acids. The highest percentage of variable amino acid positions (4.4 %) was seen in the airA and bax loci; and the lowest (0.0 %) in elfP. In the analysis of the genetic relationships of *P. salmonis* isolates, based in the analysis of concatenated HK, it were found two clades with similar grouping shown by the phylogenetic analysis of 16S rDNA-ITS genes. However, a better separation was seen in the clades G1 and G2 (Fig. 4).

**Fig. 5:**
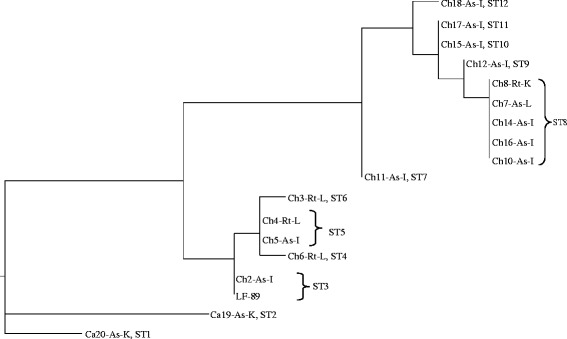
Dendrogram showing the sequence type relations in the multilocus sequence typing scheme (MLST) of the 18 isolates of *P. salmonis*. The MLST analysis was constructed using 11176nt from the HK dnaK, groEL, trpB, mltB, ospA, radA,alr, bax, tnpA, and elfP. The analysis was performed using the distance method in PAUP 4.0. The codes for all the isolates are explained in the Table 1

The MLST analyses (Fig. 5) resulted in 12 STs based on the different ATs. The Chilean isolates presented 10 STs and the Canadian, two. The MLST analysis reflects the 16S rDNA-ITS phylogeny, arranging the isolates in three groups (Fig. 5).

## Discussion

The present study shows the 18 Chilean *P. salmonis* isolates as two genetically distinct groups, a separation supported by all three genotyping methods. Clade G1, containing isolates from Puerto Montt area and two isolates from Chiloe; and clade G2, including isolates retrieved from sites along the East coast of Chiloé Island and Melinka Island (Fig. 1). The present study, which does not included European isolates, clearly shows that the 18 Chilean isolates are genetically distinct from the two Canadian isolates of *P. salmonis* (Figs. 3, 4 and 5). Previous studies have shown genetic variations among Chilean isolates of *P. salmonis* and that Chilean strains are more genetically related to each other than to European and Canadian isolates [19–21]. However, these phylogenetic studies have only consisted of five or less Chilean isolates [19, 20], and were therefore not able to have sufficient resolution to separate them in phylogenetic clades.

All three genetic methods used in this study are suitable for separation of *P. salmonis* isolates, and we present the first MLST typing system for *P. salmonis*. However, more isolates from a wider geographical area are needed for mapping of geographical distribution, reservoirs, possible transmission routes, host specificity, and variation in tissue tropism. Other methods, like variable number of tandem repeats (VNTR), giving a better separation of the isolates, are needed for studies of virulence.

The isolates studied were separated in two groups under the optimum growing temperature tests which correlate to a large extent with the results of genotyping. An optimal cultivation temperature at 16–19 °C was seen for isolates includes in clade G1 isolates confirming the findings of Fryer et al. [22]. Whereas, the less fastidious isolates, included in clade G2, had an optimal temperature between at 19–22 °C, which correlates with data presented by Mikalsen et al. [10].

The Canadian isolates differed both in genetic and phenotypic characteristics, as both were isolated from the same site during a contemporary outbreak SRS, showing that two different strains of *P. salmonis* can occur during a single outbreak of SRS.

Comparison of the different culture media clearly shows that SRS-BA is the best medium for culturing all the isolates included. Ch14-As-I had the fastest growth and was the only isolate positive for β-galactosidase and α-glucosidase in the API ZYM test. The presences of α-glucosidase and β-galactosidase have also been described to vary for strains of the related intracellular bacterium *Francisella philomiragia* subsp. *philomiragia* [23]. β-galactosidase and α-glucosidase participate in metabolic pathways in Gram negative bacteria [24] and might influence the bacterial growth.

## Conclusions

This is the first study showing that there are two genetic groups of *P. salmonis* present in Chile, and that more than one strain of *P. salmonis* can occur under a contemporary outbreak of SRS. The two Canadian isolates were genetically separate when compared to the 18 Chilean isolates, although belong to the same branch. Other isolates should be included in further genotyping studies on Canadian strains of *P. salmonis*. The genotyping methods used are suitable for separation of the isolates; however, other molecular tools are most likely needed for separating isolates with different virulence. The phenotypic tests showed variation among the isolates, with the temperature test giving the best separation. In future studies the SRS-BA should be the preferred agar for isolation and culturing of *P. salmonis*. Studies of geographical distribution and host specificity will have to include more isolates from different fish hosts in all areas with production of salmonids and presence of *P. salmonis*.

The MLST and the sequences alignments for 16S rRNA-ITS and the concatenated housekeeping genes are made available at the TreeBASE data base (http://purl.org/phylo/treebase/phylows/study/TB2:S18993).

### Availability of supporting data

The nucleotide sequences are deposited in the GenBank data Base DOI http://www.ncbi.nlm.nih.gov/genbank/. The accession numbers are presented in the Additional file 1: Table S1.

## Additional file

Additional file 1: Table S1. Accession numbers of the *P. salmonis* isolates included in the study. (DOCX 20 kb)

## Abbreviations

ANI: average nucleotide identity
DSB: defibrinated sheep blood
HK: housekeeping genes
ITS: internal transcriber space
MA: marine agar
MLST: multi locus of sequence typing
RO: reverse osmosis water
RSS: red sea salt
SRS: salmonid rickettsial septicemia
SRS-BA: salmonid rickettsial septicemia-blood agar
TSA: tryptic soy agar
VNTR: variable number of tandem repeats
