# Supplementary material for: Phenotypic and genetic characterization of Piscirickettsia salmonis from Chilean and Canadian salmonids
Source: BMC Vet Res. 2016 Mar 15;12:55. doi: 10.1186/s12917-016-0681-0 (PMC4791975; doi:10.1186/s12917-016-0681-0)
Supplement: Additional file 1: Table S1. — Accession numbers of the P. salmonis isolates included in the study. (DOCX 20 kb) [file 12917_2016_681_MOESM1_ESM.docx]

**Additional file 1**

Table S1 Accession numbers of the *P. salmonis* isolates included in the study

| Code | 16s-ITS | alr | dnaK | ospA | tbpB | radA | mltB | groEL | bax | tnpA | elfP |
| --- | --- | --- | --- | --- | --- | --- | --- | --- | --- | --- | --- |
| LF-89 | KF990218 | KJ081102 | KJ143842 | KJ143862 | KJ143882 | KJ735722 | KJ999171 | KJ735742 | KM055078 | KM055096 | KJ999152 |
| Ch2-As-I | KF990219 | KJ081103 | KJ143843 | KJ143863 | KJ143883 | KJ735723 | KJ999172 | KJ735743 | KM055079 | KM055097 | KJ999153 |
| Ch3-Rt-L | KF990220 | KJ081104 | KJ143844 | KJ143864 | KJ143884 | KJ735724 | KJ999173 | KJ735744 | KM055080 | KM055098 | KJ999154 |
| Ch4-Rt-L | KF990221 | KJ081105 | KJ143845 | KJ143865 | KJ143885 | KJ735725 | KJ999174 | KJ735745 | KM055081 | KM055099 | KJ999155 |
| Ch5-As-I | KF990222 | KJ081106 | KJ143846 | KJ143866 | KJ143886 | KJ735726 | KJ999175 | KJ735746 | KM055082 | KM055100 | KJ999156 |
| Ch6-Rt-L | KF990223 | KJ081107 | KJ143847 | KJ143867 | KJ143887 | KJ735727 | KJ999176 | KJ735747 | KM055083 | KM055101 | KJ999157 |
| Ch7-As-L | KF990224 | KJ081108 | KJ143848 | KJ143868 | KJ143888 | KJ735728 | KJ999177 | KJ735748 | KM874458 | KM055102 | KJ999158 |
| Ch8-Rt-K | KF990225 | KJ081109 | KJ143849 | KJ143869 | KJ143889 | KJ735729 | KM055077 | KJ735749 | KM055084 | KM055103 | KJ999159 |
| Ch10-As-I | KF990227 | KJ081111 | KJ143851 | KJ143871 | KJ143891 | KJ735731 | KJ999179 | KJ735751 | KM055085 | KM055104 | KJ999161 |
| Ch11-As-I | KF990228 | KJ081112 | KJ143852 | KJ143872 | KJ143892 | KJ735732 | KJ999180 | KJ735752 | KM055086 | KM055105 | KJ999162 |
| Ch12-As-I | KF990229 | KJ081113 | KJ143853 | KJ143873 | KJ143893 | KJ735733 | KJ999181 | KJ735753 | KM055087 | KM055106 | KJ999163 |
| Ch14-As-I | KF990231 | KJ081115 | KJ143855 | KJ143875 | KJ143895 | KJ735735 | KJ999183 | KJ735755 | KM055089 | KM055108 | KJ999164 |
| Ch15-As-I | KF990232 | KJ081116 | KJ143856 | KJ143876 | KJ143896 | KJ735736 | KJ999184 | KJ735756 | KM055090 | KM055109 | KJ999165 |
| Ch16-As-I | KF990233 | KJ081117 | KJ143857 | KJ143877 | KJ143897 | KJ735737 | KJ999185 | KJ735757 | KM055091 | KM055110 | KJ999166 |
| Ch17-AS-I | KF990234 | KJ081118 | KJ143858 | KJ143878 | KJ143898 | KJ735738 | KJ999186 | KJ735758 | KM055092 | KM055111 | KJ999167 |
| Ch18-As-I | KF990235 | KJ081119 | KJ143859 | KJ143879 | KJ143899 | KJ735739 | KJ999187 | KJ735759 | KM055093 | KM055112 | KJ999168 |
| Ca19-As-I | KF990236 | KJ081120 | KJ143860 | KJ143880 | KJ143900 | KJ735740 | KJ999188 | KJ735760 | KM055094 | KM055113 | KJ999169 |
| Ca20-As-I | KF990237 | KJ081121 | KJ143861 | KJ143881 | KJ143901 | KJ735741 | KJ999189 | KJ735761 | KM055095 | KM055114 | KJ999170 |
